# Neonatal intensive care nurses’ assessment of preterm infants’ pain and sedation: inter-rater reliability of the neonatal pain, agitation, and sedation scale

**DOI:** 10.3389/fped.2025.1639511

**Published:** 2026-01-12

**Authors:** Selvinaz Albayrak, Zehra Kan Öntürk, Elif Şen, Melike Yayla

**Affiliations:** 1 Department of Nursing, Faculty of Health Sciences, Istinye University, Istanbul, Türkiye; 2 Department of Nursing, Faculty of Health Sciences, Acibadem Mehmet Ali Aydinlar University, Istanbul, Türkiye; 3 Neonatal Intensive Care Unit, Acıbadem Health Group Altunizade Hospital, Istanbul, Türkiye

**Keywords:** inter-rater reliability, neonatal intensive care units, neonatal nursing, neonatal pain assessment, pain management

## Abstract

**Background:**

Timely and accurate assessment of pain and sedation in newborns is essential for effective management. Therefore, neonatal pain and sedation assessment remains a key global issue in neonatal intensive care unit (NICU) nursing practice. This study examined the inter-rater reliability of Neonatal Pain Agitation, and Sedation Scale (N-PASS) scores among NICU patients.

**Methods:**

This prospective observational study assessed agreement among 19 NICU nurses and two independent researchers who completed 190 observations from 82 preterm infants. Each evaluator rated N-PASS independently and blindly. Agreement among three raters—a nurse and two researchers—were analyzed using the intraclass correlation (ICC) and the Fleiss kappa test.

**Results:**

Agreement levels varied across N-PASS subscales. The ICC and kappa values indicated moderate-to-good reliability for the pain/agitation subscale, whereas the ICC values for the sedation subscale indicated excellent or moderate reliability. Nurses assigned higher mean pain/agitation scores than researchers.

**Conclusions:**

NICU nurses must improve their N-PASS assessment skills for both pain and sedation. NICU nurse managers should prioritize improving these competencies to improve pain experiences and ensure adequate sedation, given their significant impact on short- and long-term outcomes in preterm infants.

## 1 Introduction

Pain is common among infants in neonatal intensive care units (NICUs), where many undergo repeated diagnostic and therapeutic procedures during hospitalization (1, 2). Because frequent painful exposures and early-life stress may negatively affect physiologic stability and neurodevelopment in preterm infants, accurate recognition and management of pain remains a critical clinical priority in NICUs (3–10).

Although the importance of neonatal pain assessment is well recognized, practices across NICUs remain inconsistent (1, 11, 12). Reliable evaluation of pain, agitation, and sedation is necessary to prevent both undertreatment and unnecessary exposure to analgesic or sedative medications (13). Pain assessment is challenging due to infants' inability to communicate verbally, gestational age-related differences in behavioral cues, and variability in clinician training and experience (14–16). Furthermore, physiologic and behavioral stress responses often resemble pain-related cues, complicating differentiation in clinical settings (14). This overlap can lead to misclassification, causing either insufficient analgesia or unwarranted pharmacologic treatment (17).

Consistent use of structured assessment tools is essential to support accurate clinical decision-making (18). Nurses play a central role in applying these tools in the NICU; however, studies have shown variability in assessment accuracy (19). Several pain assessment scales exist for neonatal populations (14, 20, 21). Among these, the Neonatal Pain, Agitation, and Sedation Scale (N-PASS) is widely used because it combines behavioral and physiologic indicators and is applicable across a broad gestational age range for assessing both pain and sedation (22–24). However, interpretation of these indicators in preterm and critically ill infants may differ among assessors, making inter-rater reliability an important component of the tool's clinical validity (14). Only a limited number of studies have evaluated the inter-rater reliability of the N-PASS in real-time clinical settings (22, 23, 25).

Therefore, this study aimed to evaluate the inter-rater reliability of the N-PASS in a real clinical setting. Considering that consistency among assessors is essential for accurate pain evaluation, this study specifically examined the consistency of NICU nurses' pain and sedation assessments using the N-PASS in preterm infants.

## 2 Methods

### 2.1 Study design, setting and participants

This prospective, blinded observational study examined agreement of N-PASS scores among NICU nurses. The study followed the Strengthening the Reporting of Observational Studies in Epidemiology (STROBE) guidelines (26) and was registered in the Clinical Trial Registry of the U.S. National Institutes of Health (https://clinicaltrials.gov/; identifier: NCT06885437). All procedures adhered to the Declaration of Helsinki and were approved by the “Acıbadem Mehmet Aydınlar University Medical Research Review Board (ATADEK) (Decision no: 2022-20/25, December 30, 2022).”

Data collection took place between January and March 2023 in a 28-bed NICU staffed by 26 nurses working two 12-h shifts daily. Except for the charge nurse, all nurses rotated through both day and night shifts based on the weekly staffing schedule. Inclusion criteria required at least 6 months of NICU experience and willing to participate; nurses with <6 of experience were excluded. Before enrollment, nurses received information about the study purpose, procedures, voluntary participation, and the right to withdraw at any time. Both verbal and written informed consent were obtained. Two nurses (7.7%) declined participation, and five (19.2%) did not meet eligibility requirements, resulting in a final sample of 19 nurses (73.1%). Data were collected during day-shift assignments.

Following recommendations by Koo and Li (2016), which emphasize obtaining a heterogeneous sample of at least 30 participants and including at least three raters in reliability studies, this study included 190 observations from 82 preterm infants younger than 37 gestational weeks. Repeated observations for the same infant were allowed only when separated by at least 24 h. Inclusion criteria for infants were all preterm infants admitted during the study period; exclusion criteria were major congenital anomalies, severe neurologic disorders, or paralysis conditions that could affect accurate pain assessment. Pain and sedation assessments from 190 observations were completed simultaneously by three raters—two researchers and one nurse. Each of the 19 nurses assessed 10 infants, yielding a total of 190 infant assessments (19 nurses × 10 infants). Thus, two researchers and one nurse evaluated each infant together, consistent with rater-based clinical assessment methods described on the literature (27).

The N-PASS scale has been routinely used in the NICU since 2020 to assess pain and agitation 6 times daily for all infants, and sedation for those receiving sedative medications. At implementation, nurses completed structured training covering key principles of neonatal pain assessment and practical scoring procedures. Newly hired nurses received the same orientation during onboarding. Therefore, all raters in this study were familiar with the N-PASS and competent in its routine clinical application.

### 2.2 Instruments

#### 2.2.1 Nurse demographics and training for pain assessment and management

This section included six research-developed questions covering nurses' age, sex, educational status, total professional experience, NICU experience, and prior training in neonatal pain and sedation assessment and management.

#### 2.2.2 Infant demographics

This section included 10 research-developed questions regarding each infant's sex, gestational age at birth, birth weight, current weight, diagnosis, type of ventilation support, sedation interventions, and pain relief interventions.

#### 2.2.3 Neonatal pain agitation and sedation scale (N-PASS)

The N-PASS, developed by Hummel et al. (2008), consists of two subscales, with pain/agitation and sedation scores calculated separately (22, 24, 28, 29). Each subscale includes four behavioral items—crying or irritability, behavior state, facial expression, and extremities tone—and one vital signs item assessing changes in heart rate, respiratory rate, blood pressure, and oxygen saturation (25). The N-PASS is used for premature infants, for assessing acute and chronic pain, and for infants receiving mechanical ventilation. It has been validated for infants from 23 gestational weeks to 36 months, making its suitable for preterm infants in this study, including those born at 24 weeks. Pain and agitation items are scored as 0, 1, or 2 (22, 25). “The maximum score is 11 for preterm infants younger than 30 weeks and 10 for those older than 30 weeks. Pain levels are categorized as no pain (0–3), mild pain (4–7), and severe pain (8–11)” (22, 30). Sedation scoring uses the same behavioral and physiologic categories, scored as 0, −1, or −2. The maximum is −10, with 0 indicating no sedation. Score from −1 to −5 reflect light sedation, and scores from −5 to −10 represent deep sedation (25). Cronbach's alpha coefficients for the original pain and sedation subscales were 0.82 and 0.87, respectively (22, 25). The Turkish adaptation by Açıkgöz et al. (2017) reported Cronbach's alpha values were 0.83 and 0.77 for the sedation subscale (31). In this study, Cronbach's alpha values were 0.89 for the pain subscale and 0.95 for the sedation subscale. Other studies using the Neonatal Pain, Agitation, and Sedation Scale reported Cronbach's alpha values of 0.93, 0.84, and 0.85 for the pain subscale (32–34) and 0.88 and 0.73 for the sedation subscale (28, 34). Overall, the N-PASS scale is a reliable tool for assessing acute pain in both mechanically and nonmechanically ventilated neonates, evaluating long-term pain in mechanically ventilated or postoperative infants, and determining sedation levels in mechanically ventilated or postoperative infants (29).

### 2.3 Data collection

Data collection was performed by two researchers and NICU nurses. Both researchers held Bachelor of Science in Nursing degrees, had 5 years of NICU experience, and were certified NICU nurses. One researcher served as a charge nurse and the other as a clinical nurse educator.

Each observation involved two fixed researchers and one bedside nurse. The two researchers evaluated all infants throughout the study, whereas the bedside nurse rotated according to shift assignments. Thus, each assessment was independently completed by a three-rater team: two consistent researchers and one rotating bedside nurse.

After identifying infants who met the inclusion criteria, the N-PASS scores for pain, agitation, and sedation were independently and blindly assessed by the three observers. The two researchers completed 190 assessments, and each of the 19 nurses scored 10 preterm infants. Pain and agitation assessments were performed after routine nursing care (monitoring vital signs, feeding support, positioning and comfort care, hygiene, and skin care) in 190 observations from 82 preterm infants. Sedation assessments were conducted after routine care in 46 preterm infants receiving sedatives. All assessments were completed simultaneously at the bedside through direct observation.

Each observer was given one minute to independently record the total Neonatal Pain, Agitation, and Sedation Scale score. Demographic and clinical data for both nurses and infants were also documented. Blinding procedures were strictly followed. Each observer independently evaluated the same infant without access to the other raters' scores. Observers recorded their assessments on separate data collection forms at the bedside without any verbal or visual communication about their evaluations. Nurses and researchers remained blinded to one another's ratings and submitted their completed forms individually to the third researcher at the end of data collection.

### 2.4 Data analysis

There were no missing data. A total of 190 observations for pain and agitation and 46 observations for sedation were analyzed using Statistical Package for the Social Sciences version 26.0 (IBM, Armonk, NY, USA). Descriptive statistics were used to summarize the demographic characteristics of nurses and infants, as well as the N-PASS scores. Skewness and kurtosis values were examined to assess the normality of summed pain, agitation, and sedation scores. Inter-rater reliability was evaluated by comparing the three raters' N-PASS pain and sedation scores. Intraclass correlation coefficient (ICC) values were calculated for both single and average measures. A two-way random-effects model was selected because it is appropriate for rater-based clinical assessment methods. Absolute agreement was used to determine whether different raters assigned identical values. ICC values were ICC values were interpreted as follows: <0.50, poor reliability; 0.50–0.75, moderate reliability; 0.75–0.90, good reliability; and greater than 0.90, excellent reliability (27). The paired t-test was used to compare pain/agitation scores across the three raters. The Wilcoxon signed-rank test was used to compare sedation scores because of the smaller sample size.

N-PASS pain scores were categorized as no pain (0–3), mild pain (4–7), and severe pain (8–11). Sedation scores were categorized as “no sedation (0), light sedation (−1 to −5), and deep sedation (−6 to −10). Fleiss kappa statistics with absolute agreement were used to assess the inter-rater reliability of the three observers for these categorical variables. Kappa (*κ*) values were interpreted as follows: none (0–0.20), minimal (0.21–0.39), weak (0.40–0.59), moderate (0.60–0.79), strong (0.80–0.90), and almost perfect (greater than 0.90) (35). Bland–Altman plots were generated to analyze agreement in pain, agitation, and sedation scores among raters. Finally, Spearman correlation analysis was performed to examine relationships between pain, agitation, and sedation scores assigned by each rater, given that sedative use may suppress behavioral pain indicators without providing analgesia (22).

## 3 Results

### 3.1 Demographic characteristics of nurses

Nineteen NICU nurses participated in the study. Their characteristics are summarized in Table 1. The mean duration of NICU experience was 3.37 ± 2.19 years.

**Table 1:** Characteristics of NICU nurses (*N* = 19).

Variables of nurses	Mean (SD)	Min–Max (Median)
Age (years)	23.95 (1.65)	20–26 (24)
Duration of experience working in existing NICU (years)	3.37 (2.19)	1–6 (3)
Duration of total professional experience as NICU nurse (years)	4.58 (3.13)	1–7 (4)
Duration of experience using N-PASS scale	2.19 (0.61)	1–3 (2)
Sex	*n*	%
Female	18	94.7
Male	1	5.3
Educational level		
High school	6	31.6
Associate degree	2	10.5
Bachelor's degree	11	57.9

### 3.2 Descriptive and clinical data of preterm infants

Descriptive and clinical characteristics of preterm infants are shown in Table 2. Most infants were male (57.4%). The mean gestational age was 30.86 ± 3.69 weeks, and the mean birth weight was 1,632.22 ± 636.29 g. Nearly half of the infants (48.9%) received respiratory support, and approximately one-fourth (24.2%) received sedation. In the previous 12 h, almost half of the infants (48.4%) received either pharmacologic or nonpharmacologic pain relief interventions.

**Table 2:** Demographic and clinical variables of preterm infants (*N* = 190).

Variables	Mean (SD)	Min–Max (Median)
Gestation week	30.86 (3.69)	24–36 (31.5)
Birth weight	1,623.22 (636.29)	530–3,010 (1,607.50)
Current weight	2033.07 (703.54)	530–4,900 (1,925)
Sex	*n*	%
Girl	81	42.6
Boy	109	57.4
Medical diagnosis		
Prematurity	162	85.3
Prematurity + respiratory distress + Apnea	18	9.5
Prematurity + bronchopneumonia	3	1.6
Prematurity + asphyxia	2	1.1
Prematurity + omphalitis	1	0.5
Prematurity + hyperbilirubinemia	1	0.5
Prematurity + sepsis + COVID-19	1	0.5
Prematurity + thrombocytopenia	1	0.5
Prematurity + fever	1	0.5
Receiving respiratory support		
Yes	93	48.9
No	97	51.1
Type of respiratory support (*n* = 93)		
HFO + intubation	34	36.5
Supplementation within the incubator	33	35.5
NCPAP	18	19.4
CPAP	8	8.6
Receiving sedation		
Yes	68	35.8
No	122	64.2
Interventions to reduce pain in the last 12 h		
Implemented	92	48.4
Not Implemented	98	51.6
Interventions for pain (*n* = 92)		
Nonpharmacologic methods		
Breast-feeding	21	22.8
Pacifier + position	18	19.6
Kangaroo	13	14.1
Pacifier + wrapping	11	12.0
Toot sweet	11	11.9
Positioning	9	9.8
Reduce touching	3	3.3
Touch + singing	2	2.2
Pharmacologic methods	4	4.3

### 3.3 Descriptive data for N-PASS

Descriptive statistics for the N-PASS are presented in Table 3. Among the 190 observations from 82 preterm infants, the mean pain and agitation scores was 3.00 ± 2.10) for the NICU nurses, 2.31 ± 1.38 for Researcher 1, and 2.29 ± 1.35 for Researcher 2. For the 46 infants who received sedation, the mean sedation scores were −1.52 ± 2.63 for the NICU nurses, −1.46 ± 2.87 for Researcher 1, and −1.37 ± 2.78 for Researcher 2. Regarding pain assessments, 65.3% of the nurses rated preterm infants' pain as within an acceptable range. In contrast, both researchers rated 80% of the infants as having acceptable pain levels and 20% as having moderate pain. Among sedated infants, 71.9% of the nurses, 73.9% of rating from Researcher 1, and 84.8% of rating from Researcher 2 assigned a sedation score of 0.

**Table 3:** Descriptive and comparison statistics of neonatal pain, agitation, and sedation scale scores by NICU nurses and two researchers.

N-PASS	Raters	Mean	SD	Median	Min/max	Skewness	Kurtosis
Pain -Agitation (*N* = 190)	Nurse	3.00	2.10	3	0/11	1.07	1.07
	Researcher 1	2.31	1.38	2	0/6	0.60	−0.46
	Researcher 2	2.29	1.35	2	0/6	0.67	−0.38
Paired sample t-test	Nurse–Researcher 1: *t* = 5.839, df = 189, *p* < 0.001						
	Nurse–Researcher 2: *t* = 5.767, df = 189, *p* < 0.001						
	Researcher 1–Researcher 2: *t* = 0.589, df = 189, *p* = 0.557, *p* > 0.05						
Sedation (*n* = 46)	Nurse	−1.52	2.63	0	0/−8	−1.42	0.52
	Researcher 1	−1.46	2.87	0	0/−10	−2.02	3.27
	Researcher 2	−1.37	2.78	0	0/−10	−2.20	4.18
Wilcoxon signed-ranks test	Nurse–Researcher 1: *z* = −0.401, *p* = 0.688, *p* > 0.05						
	Nurse–Researcher 2: *z* = −0.581, *p* = 0.562, *p* > 0.05						
	Researcher 1–Researcher 2: *z* = −1.342, *p* = 0.180, *p* > 0.05						
Pain -Agitation (*N* = 190)	Severity of pain	0–3 point (no pain)		4–7 point (mild)		8–11 point (severe)	
		*n*	%	*n*	%	*n*	%
	Nurse	124	65.3	59	31.1	7	3.7
	Researcher 1	152	80.0	38	20.0	-	-
	Researcher 2	152	80.0	38	20.0	-	-
Sedation (*n* = 46)	Level of sedation	0 point (no sedation)		−1 to − 5 point (light)		−6 to − 1 0 point (deep)	
		*n*	%	*n*	%	*n*	%
	Nurse	33	71.7	8	17.4	5	10.9
	Researcher 1	34	73.9	8	17.4	4	8.7
	Researcher 2	39	84.8	4	8.7	3	6.5

### 3.4 Agreement level for N-PASS scores

The ICC among the three raters was evaluated using a two-way random-effects model with absolute agreement. Results are presented in Table 4. The single-measure ICC for N-PASS pain and agitation scores was 0.64 (95% CI, 0.57–0.71; *F* = 6.430; *p* < 0.001), and the average-measure ICC was 0.84 (95% CI, 0.80–0.88; *F* = 6.430; *p* < 0.001). For sedation scores among the 46 preterm infants receiving sedatives, the single-measure ICC was 0.84 (95% CI, 0.76–0.90; *F* = 16.812; *p* < 0.001) and the average-measure ICC was 0.94 (95% CI, 0.90–0.97; *F* = 16.812; *p* < 0.001).

**Table 4:** Intraclass correlation among raters for the total mean N-PASS pain, agitation, and sedation scores.

N-PASS pain, agitation scores (*N* = 190)	ICC	95% confidence interval		*F* test with true value 0			
		LB	UB	F	df1	df2	*p*
Single measure	0.64	0.57	0.71	6.430	189	378	<0.001
Average measure	0.84	0.80	0.88	6.430	189	378	<0.001
N-PASS Sedation Scores (*n* = 46)	ICC	95% confidence interval		*F* test with true value 0			
		LB	UB	F	df1	df2	*p*
Single measure	0.84	0.76	0.90	16.812	45	90	<0.001
Average measure	0.94	0.90	0.97	16.812	45	90	<0.001

ICC, intraclass correlation; df, degree of freedom; LB, lower bound; UB, upper bound.

Agreement levels for N-PASS pain subscale categories among the NICU nurses and the two researchers were analyzed using Fleiss kappa statistics (Table 5). The kappa values showed weak agreement for the no-pain category (*κ* = 0.49; 95% CI, 0.49–0.50; *F* = 11.783; *p* < 0.001) and the mild-pain category (*κ* = 0.44; 95% CI, 0.43–0.44; *F* = 10.434; *p* < 0.001). No agreement was observed for the severe-pain category (*κ* = 0.01; 95% CI, −0.02 to −0.01; *F* = 0.767), and this result was not statistically significant (*p* > 0.05). Because severe pain was rare in this sample, the kappa estimate for this category was statistically unstable and its CI should not be considered a reliable indicator of agreement.

**Table 5:** Inter-rater reliability of N-PASS pain and sedation scores using Fleiss Kappa.

N-PASS pain, agitation scores (*N* = 190)^a^	Conditional probability	Kappa	SE	*z*	*p*	95% confidence interval	
Severity of pain						LB	UB
Agreement for “0–3 point (no pain)”	0.75	0.49	0.04	11.783	<0.001	0.49	0.50
Agreement for “4–7 point (mild)”	0.24	0.44	0.04	10.434	<0.001	0.43	0.44
Agreement for “8–11 point (severe)”	0.01	−0.01	0.04	−0.297	0.767	−0.02	−0.01
Overall agreement		0.45	0.04	11.280	<0.001	0.44	0.45
N-PASS sedation scores (*n* = 46)^b^	Conditional probability	Kappa	SE	*z*	*p*	95% confidence interval	
Level of sedation						LB	UB
Agreement for “0 point (no sedation)”	0.77	0.76	0.09	8.880	<0.001	0.75	0.76
Agreement for “1 to −5 point (light)”	0.15	0.53	0.09	6.252	<0.001	0.53	0.54
Agreement for “−6 to −10 point (deep)”	0.09	0.45	0.09	5.314	<0.001	0.45	0.46
Overall agreement		0.62	0.07	9.397	<0.001	0.61	0.62

LB, lower bound; UB, upper bound.

a Sample data contains 190 effective subjects and 3 raters.

b Sample data contains 46 effective subjects and 3 raters.

For sedation categories, moderate agreement was observed for the *no-sedation* group (*κ* = 0.76; 95% CI, 0.75–0.76; *F* = 8.880; *p* < 0.001). Weak agreement was found for the light-sedation group (*κ* = 0.53; 95% CI, 0.53–0.54; *F* = 6.252; *p* < 0.001) and the deep-sedation group (*κ* = 0.45; 95% CI, 0.45–0.46; *F* = 5.314; *p* < 0.001).

### 3.5 Bland–Altman graphics

**Figure 1:**
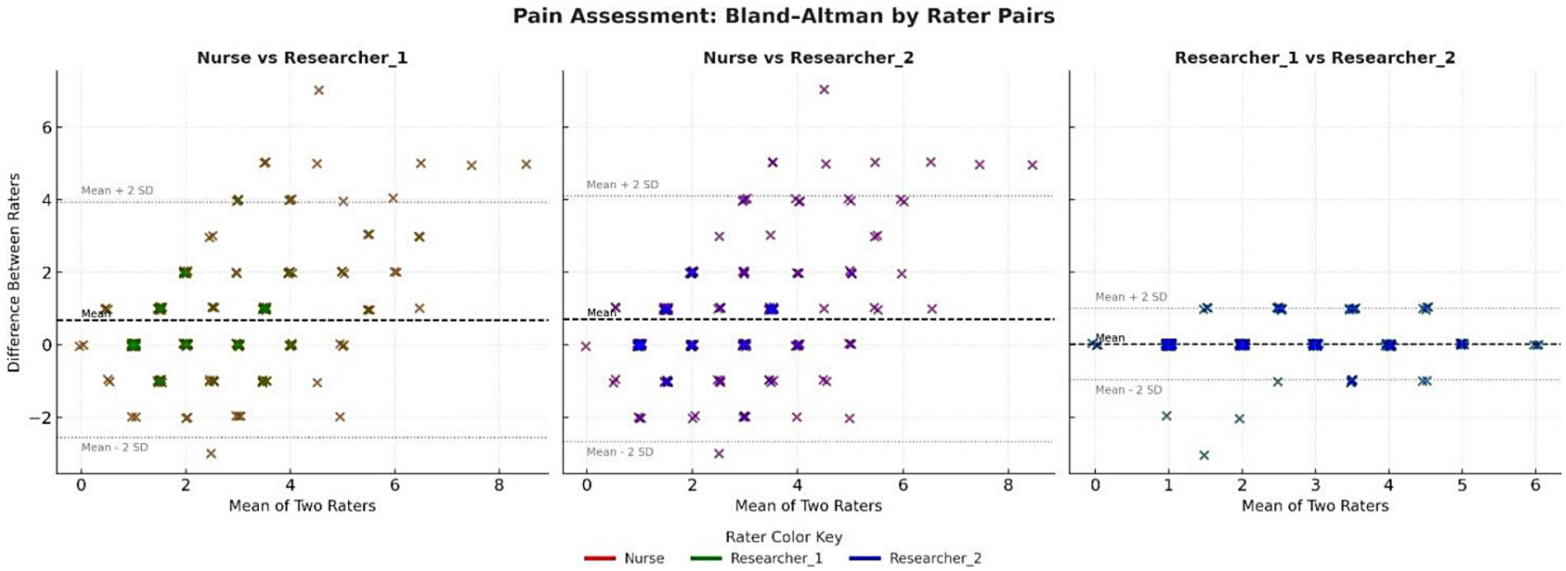
Bland–Altman graph by rater pairs for pain/agitation.

**Figure 2:**
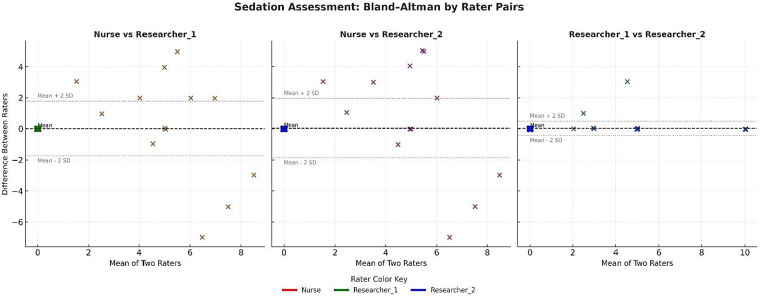
Bland–Altman graph by rater pairs for sedation.

Bland–Altman plots for the pain/agitation and sedation subscales are shown Figures 1, 2. The average differences between the nurse and both researchers were small. Most score differences fell within the 95% CI, indicating measurement consistency within clinically acceptable limits for pain/agitation assessments. The Bland–Altman analysis for sedation scores also demonstrated extremely strong agreement among the raters.

### 3.6 Correlations between each rater's pain/agitation and sedation scores

Spearman correlation analyses were conducted to examine the relationships between each rater's pain/agitation and sedation scores for individual preterm infants, and the results are presented in Supplementary Table S1. Across all 190 observations from 82 preterm infants, the correlation coefficients between pain/agitation and sedation scores were *r* = −0.03 (*p* > 0.05) for nurses, *r* = −0.14 (*p* < 0.05) for Researcher 1, and *r* = −0.13 (*p* > 0.05) for Researcher 2. Among infants who received sedation, the correlation coefficients were *r* = −0.01 (*p* > 0.05) for nurses, *r* = −0.31 (*p* < 0.05) for Researcher 1, and *r* = −0.24 (*p* > 0.05) for Researcher 2. For infants who received analgesic medications or nonpharmacologic interventions, the correlation coefficients were *r* = −0.01 (*p* > 0.05) for nurses, *r* = −0.22 (*p* < 0.05) for Researcher 1, and *r* = −0.18 (*p* > 0.05) for Researcher 2.

## 4 Discussion

This study evaluated the inter-rater reliability of the N-PASS in a real-time clinical environment. The findings provide insight into how consistently pain/agitation and sedation scores are applied by different assessors during routine NICU care.

The findings of this study revealed that inter-rater reliability for the pain/agitation subscale was moderate based on a single measurement (ICC = 0.64) but improved to “good” when averaged across raters (ICC = 0.84). These results indicate that pain assessment is subject to observer interpretation; however, scoring consistency may improve with the number of raters. Our results are lower than those reported in existing literature, which consistently indicates higher reliability. Previous research has consistently exhibited higher inter-rater reliability for the N-PASS than our real-time clinical assessments. Hummel et al. (2008) reported ICC values >0.90 under structured conditions with trained nurses, and Hummel (2017) similarly found reliability values between 0.86 and 0.93 in preterm and older infants (22, 25). Video-based assessments by Huang and Kappesser also demonstrated excellent reliability (ICC >0.90) during both painful and nonpainful procedures (32, 34). More recently, Benbrook et al. (2023) reported good reliability (ICC = 0.83–0.85) among bedside nurses following structured N-PASS training (23). The higher reliability observed in these studies likely reflects methodological differences, such as the use of trained raters, controlled environments, and standardized assessment protocols. These factors differ substantially from the real-time bedside conditions of the present study.

In this study, the kappa analysis results for inter-rater reliability on the pain/agitation subscale revealed poor agreement in the “no-pain” (*κ* = 0.49) and “mild-pain” (*κ* = 0.44) categories and no agreement in the “severe-pain” category (*κ* = –0.01). Therefore, raters expressed differing opinions when identifying severe pain in infants. As seen in Table 3 and Figure 1, nurses consistently assigned higher pain scores than the researchers, particularly in the mild-to-moderate range, which contributed to poor kappa agreement in these categories. Cignacco et al. (2008) reported that nurses perceived procedures performed on infants as more painful than physicians did (36). Such overestimation may increase the likelihood of unnecessary pharmacologic interventions, whereas underestimation may lead to insufficient analgesia, both of which have important clinical implications and may hinder opioid-sparing strategies in the NICU (37, 38). This variability in pain scoring also carries therapeutic implications (39).

Interpreting kappa coefficients in this study requires caution due to the highly unbalanced distribution of pain categories. The near absence of infants rated with severe pain created a sparse-data problem known to lower kappa scores and produce mathematically unstable estimates. This imbalance also explains the artificially narrow CIs, which result from low variance rather than high measurement precision. Therefore, lower kappa values in this context do not necessarily indicate true disagreement among raters but rather reflect a statistical limitation caused by categorical distribution. Under these conditions, ICC values derived from continuous N-PASS scores offer a more accurate representation of inter-rater reliability.

Considering that pain assessment is a core parameter evaluated at least every 4 h in the NICU, consistent scoring by bedside nurses is essential to guide appropriate pharmacologic and nonpharmacologic interventions (40). Therefore, strengthening training and standardization efforts may enhance inter-rater reliability and support safer, more effective pain management practices in preterm infants.

The findings of this study indicated good reliability (ICC = 0.84) for single measures and excellent reliability (ICC = 0.94) for average measures on the sedation subscale, which are consistent with previous research results (23, 25). Furthermore, in the study conducted by (23), nurses reported that the N-PASS effectively assessed neonatal sedation status at a “good” or “very good” level. The findings of this study revealed good reliability for single measures (ICC = 0.84) and excellent reliability for average measures (ICC = 0.94) on the sedation subscale, consistent with previous research (23, 25). In the present analysis, the mean kappa value among three raters indicated moderate agreement, and Figure 2 demonstrates a close distribution of sedation scores between the nurses and researchers, in contrast to the wider dispersion observed in pain scoring. This pattern may reflect that sedation cues tend to be interpreted more consistently than pain-related behaviors.

Another notable finding in this study is that researchers identified a negative correlation between pain/agitation and sedation scores for the same patient, a finding consistent with known pharmacologic effects where sedatives may reduce observable pain behaviors and analgesics may contribute to mild sedation (22). In contrast, this relationship was not evident in nurse-assigned scores, particularly among infants receiving sedatives or analgesics. Furthermore, nurses showed less consistency when rating values above zero, often assigning higher sedation scores. These discrepancies suggest that distinguishing overlapping pain- and sedation-related cues is more challenging during routine bedside care, whereas investigator assessments exhibited a more consistent scoring pattern.

Although nurse characteristics could not be examined separately in this study, previous research indicates that demographic factors and clinical experience do not account for variability in pain scoring and that standardized education and structured approaches are more influential in improving inter-rater consistency (36).

Limitations of this study include: 1) First, the study's single-center conduct may be a limitation. However, the participation of approximately three-quarters of the nurses working on the unit, the fact that raters assessed pain and sedation in real clinical setting, and the selection of an appropriate method for ICC analysis may support the study's generalizability. Second, because data collection occurred in a real clinical setting and researchers faced time constraints, all data were gathered only during the day shift, which may have introduced selection bias. A multicenter design and data collection across both day and night shifts are recommended for future studies. Third, although daytime-only data collection is not expected to substantially affect sample representativeness, a minor possibility of selection bias cannot be entirely ruled out.

## 5 Conclusion

This prospective, blinded observational study examined the inter-rater agreement of the N-PASS. In total, 190 observations from 82 preterm infants were evaluated by two researchers and 19 NICU nurses. The findings demonstrated varying levels of agreement across N-PASS subscales. The ICC values indicated moderate-to-good reliability for the pain/agitation subscale and excellent reliability for the sedation subscale. Consistent with the ICC results, the Kappa analysis showed lower agreement for pain/agitation but higher agreement for sedation. Notably, nurses assigned significantly higher pain scores than the researchers.

To reduce this variability and strengthen clinical care, a multidisciplinary approach emphasizing enhanced education is recommended. NICU nurses should focus on both theoretical knowledge and practical skill development through innovative training methods, such as simulation or video-based calibration. Furthermore, collaborative refinement of pain and sedation assessment and management protocols is essential to promote consistency and alignment across all healthcare disciplines in the NICU.

## Data Availability

The raw data supporting the conclusions of this article will be made available by the authors, without undue reservation.
